# Associations of peripheral blood lymphopenia to disease course, treatment and TNF-α in sarcoidosis

**DOI:** 10.1186/s12931-025-03212-x

**Published:** 2025-04-09

**Authors:** Avinash Padhi, Anders Eklund, Clas Malmeström, Elina Erikson, Gustav Hallén, Anna Smed-Sörensen, Susanna Kullberg

**Affiliations:** 1 Division of Immunology and Respiratory Medicine, Department of Medicine Solna, Center for Molecular Medicine, Karolinska Institutet, Stockholm, Sweden; 2 Department of Respiratory Medicine, Theme Inflammation and Ageing, Karolinska University Hospital, Gävlegatan 55, NB3:03, 17176 Stockholm, Sweden; 3 Laboratory of Immunology, Sahlgrenska University Hospital, Gothenburg, Sweden; 4 Department of Clinical Immunology and Transfusion Medicine, Karolinska University Hospital, Stockholm, Sweden; 5 Department of Medicine, Huddinge, Center for Hematology and Regenerative Medicine, Karolinska Institutet, Stockholm, Sweden; 6 Department of Clinical Chemistry, Karolinska University Hospital, Stockholm, Sweden; 7 Department of Cell and Molecular Biology, Karolinska Institutet, Stockholm, Sweden

**Keywords:** Sarcoidosis, Lymphopenia, Treatment, TNF-α, B-cells, T-cells, NK cells

## Abstract

**Background:**

Severe sarcoidosis has been associated with peripheral blood (PB) total lymphopenia and high tumour necrosis factor α (TNF-α) levels, and the lymphopenia phenotype seems to respond poorly to conventional treatment. However, the mechanisms behind PB lymphopenia and its correlation with TNF-α levels remain unclear. Understanding the connections among PB lymphocyte subsets, TNF-α and clinical phenotype including treatment status could offer insights into how to individualize therapy.

**Methods:**

PB samples from 65 consecutive sarcoidosis patients were collected at the Department of Respiratory Medicine, Karolinska University Hospital. Total lymphocyte, T-, B- and natural killer cell and TNF-α serum concentrations were measured and correlated to clinical parameters. Penias were defined as values below the lower limit of normal. The medical charts were retrospectively searched for the first PB total lymphocyte count, mostly recorded at time around diagnosis.

**Results:**

PB total lymphopenia was observed in 35% of patients, was present since time around diagnosis, and associated with a need for treatment later (*p* = 0.005). Lymphocyte counts did not change by therapy, except for an increase in patients receiving TNF-α inhibitors (TNFi) (*p* < 0.05).

B-cell penia, observed in 37% of patients, was the most common abnormality, also in patients with normal total lymphocyte counts, while T-cell penia mainly occurred in patients with total lymphopenia (91 vs 5%, *p* < 0.001).

**Conclusions:**

B-cell penia is common in sarcoidosis patients while T-cell penia is mainly a feature of the sarcoidosis PB lymphopenia phenotype. Increased lymphocyte counts during TNFi treatment suggests that TNF-α signaling is of importance for sarcoidosis associated lymphopenia.

**Supplementary Information:**

The online version contains supplementary material available at 10.1186/s12931-025-03212-x.

## Background

The clinical presentation of the inflammatory systemic disease sarcoidosis is variable. Virtually any organ can be affected, but the lungs and/or intrathoracic lymph nodes are engaged in most cases. Patients with Löfgren’s syndrome (LS) experience an acute and often self-limiting disease, while patients with non-Löfgren’s syndrome (non-LS) more often present with a slower developing and non-resolving disease. There is no cure and despite undergoing treatment, many patients disclose a progressive disease course leading to organ function impairments and sometimes failure [1]. High release of tumour necrosis factor α (TNF-α) from macrophages was observed in patients with progressive and corticosteroid resistant disease and high serum levels associates with severe disease [2, 3]. Peripheral blood (PB) lymphopenia is reported in up to 40% of sarcoidosis patients [4] and associates with less favorable prognosis and non-response to 1st and 2nd line treatments with corticosteroids (CS) and methotrexate (MTX) [5–8], but the underlying mechanisms for these observations are poorly understood. Dysfunctional T regulatory cells (T_regs_) with an inability to suppress excessive secretion of TNF-α but with a retained anti-proliferative capacity is one suggested mechanism that explains the PB lymphopenia [9]. Migration of PB lymphocytes to the lung/other organs [8, 10] and lymphocyte depletion/increased turnover [11, 12] are other suggested explanations. Few studies have focused on the various PB lymphocyte subsets, but findings so far indicate a general lymphopenia, at least involving both CD4+ and CD8+ T-cells as well as B-cells [8, 13, 14]. Natural killer (NK) cells are lymphocytes expressing CD56 but not CD3 (CD3− CD56+) and have the capacity to secrete pro-inflammatory cytokines including TNF-α. Results on PB NK cells in sarcoidosis are contradictory. An increased frequency and cytotoxic activity have been reported [15], while other studies did not detect any difference in frequency, and a lower activity compared to healthy controls [16–18]. Mapping of PB lymphopenia and lymphocyte subsets in relation to TNF-α levels, clinical parameters including treatment regimens may help to disentangle immunological mechanisms behind and provide clues for individualized sarcoidosis treatment.

## Methods

### Study subjects

Consecutive patients with a sarcoidosis diagnosis at the outpatient clinic, Department of Respiratory Medicine, Karolinska University Hospital, Stockholm Sweden, were included by one of the authors (SK) between June 2022 to February 2023. Sarcoidosis diagnosis was established according to criteria outlined by the World Association of Sarcoidosis and other Granulomatous Disorders (WASOG) [19]. Patients with current or previous hematological disease, ongoing infection and those on treatment for an autoimmune disease were excluded. All included patients had signed a written consent form according to Declaration of Helsinki, and approval was granted from the regional ethical review board.

### Study design

PB samples, including measurement of total lymphocyte concentration and serum angiotensin-converting enzyme (s-ACE) were obtained as part of the normal clinical follow-up. The patients underwent extra sampling solely for the purpose of this study for analysis of PB concentrations and percentages of T-, B- and NK cells and serum tumour necrosis factor α (s-TNF-α). The analyses were performed at Karolinska University Laboratory or affiliated accredited external laboratories. For s-TNF-α analysis, samples were sent to Laboratory of Immunology, Sahlgrenska University Hospital Gothenburg.

The medical charts were retrospectively searched for the first PB total lymphocyte value available, mostly recorded at referral. Patients were divided into 2 groups; one consisting of patients with PB total lymphopenia and one with normal PB lymphocyte counts. Data on sex, age at sarcoidosis diagnosis, extra pulmonary manifestations (EPM) and information about treatment were also extracted. The treatments were categorized as follows; single corticosteroid (CS), cytotoxic (i.e. MTX or azathioprine) ± CS, TNF-α inhibitor (TNFi, i.e. infliximab) + CS/MTX. Lung function parameters and chest X-ray staging according to Scadding (0 = no visible intrathoracic pathology compatible with sarcoidosis, I = lymphadenopathy, II = parenchymal infiltrates and lymphadenopathy, III = parenchymal infiltrates, IV = fibrosis) [20] were matched as close as possible to the date of inclusion. EPM was defined as a positive biopsy from the affected organ or obvious symptoms/assessment from a specialist in the area. Resolving disease was defined as no signs of sarcoidosis, evaluated by chest X-ray, lung function test, absence of EPM, patient symptoms and laboratory signs of inflammation. Stable disease was defined as remaining pulmonary manifestations without deterioration, no signs of inflammatory activity in laboratory parameters and no systemic treatment required. In analysis patients with stable and resolving disease were grouped together. Patients with worsening pulmonary manifestations and/or EPM, signs of increasing inflammatory activity in laboratory parameters and/or in need of systemic treatment were classified as having an active disease.

### Analysis of PB lymphocytes

PB was collected in K_2_EDTA-tubes and analyzed within 24 h. Total white blood cell and lymphocyte counts were determined using an automated hematology analyzer (Sysmex XN, Sysmex, Japan). The reference value for lymphocytes was derived from a cohort of 833 healthy adults and follows the recommendation from the Swedish external quality control organization, EQUALIS [21, 22]. Total PB lymphopenia at inclusion was defined as results below the lower limit of normal using laboratory reference values; 1.1–3.5 × 10^9^/liter (l) and 0.8–4.1 × 10^9^/l for one patient. This patient was excluded when calculating medians. As the reference value for PB lymphocyte changed over the years, it was in many cases different between the value at inclusion and the very first value recorded, retrospectively retrieved from the medical chart. For the very first recorded value, lymphopenia was defined as <1.0 × 10^9^/l for 36, <1.1 × 10^9^/l for 23 and <0.8 × 10^9^/l for 6 patients (reference values 1.0–4.0, 1.0–3.0, 1.1–3.5 and 0.8–4.1 × 10^9^/l). Therefore, when comparing the values at inclusion with the very first retrospective values, percentage of lower normal limit was used for calculation, a method previously used [23].

### Analysis of PB lymphocyte subsets

PB was collected in an EDTA tube, kept at room temperature and analyzed within 24 h on a Navios or Aquios CL flow cytometer (Beckman Coulter). T-, B- and NK cells were counted in whole blood using flow cytometry techniques following standard protocols with fluorescently labeled antibodies. After identification of CD45+ lymphocytes using Kaluza C analysis software, the following gating strategy was applied: B cells: CD3–CD19+, T cells: CD3+, NK cells: CD3–CD16+56+. Normal ranges for the cell populations (concentrations and percentages) were derived from the healthy donor cohort used to standardize the assay. Penias were defined as results below the lower limit of normal concentrations and percentages for CD3+ (0.65–1.57 × 10^9^/l and 59–83%), CD3–CD19+ (0.08–0.28 × 10^9^/l and 6–17%) and CD16/56+ cells (0.1–0.35 × 10^9^/l and 6–26%). In the following text, penia refers to concentrations (counts) if not clearly stated otherwise.

### Analysis of TNF-α

PB was collected in a serum tube, centrifugated and frozen. S-TNF-α was determined using a chemiluminiscense method (Immulite® 1000, Siemens), a polystyrene beadbased system according to laboratory standard operating protocol. Level of detection (LOD) was <4 pg/ml. Upper limit of quantification ULOQ was 1000 pg/ml. Reference value (<20 pg/ml) was derived from a healthy blood-donor cohort.

### Statistical analysis

All statistical analysis were performed using PRISM (version 10, GraphPad software, San Diego, CA) and open-statistical platform Jamovi 1.1.9.0 (https://www.jamovi.org). Descriptive statistics were used for calculating median values and 25th-75th percentile. Data was presumed to have a non-standard distribution and comparison between groups were performed using Kruskal–Wallis test, Mann–Whitney and Fisher’s exact tests and 2 way ANNOVA model in the presence of repeated measures. Comparison between paired samples was performed using Wilcoxon rank. Correlation between parameters was performed using Spearman’s correlation. *P*-values <0.05 were considered statistically significant and denoted by ^∗^*p* < 0.05; ^∗∗^*p* < 0.01; ^∗∗∗^*p* < 0.001; and ^∗∗∗∗^*p* < 0.0001.

S-TNF-α below detection level of 4 was calculated as 0 in analysis.

## Results

### Study subjects

**Fig. 1:**
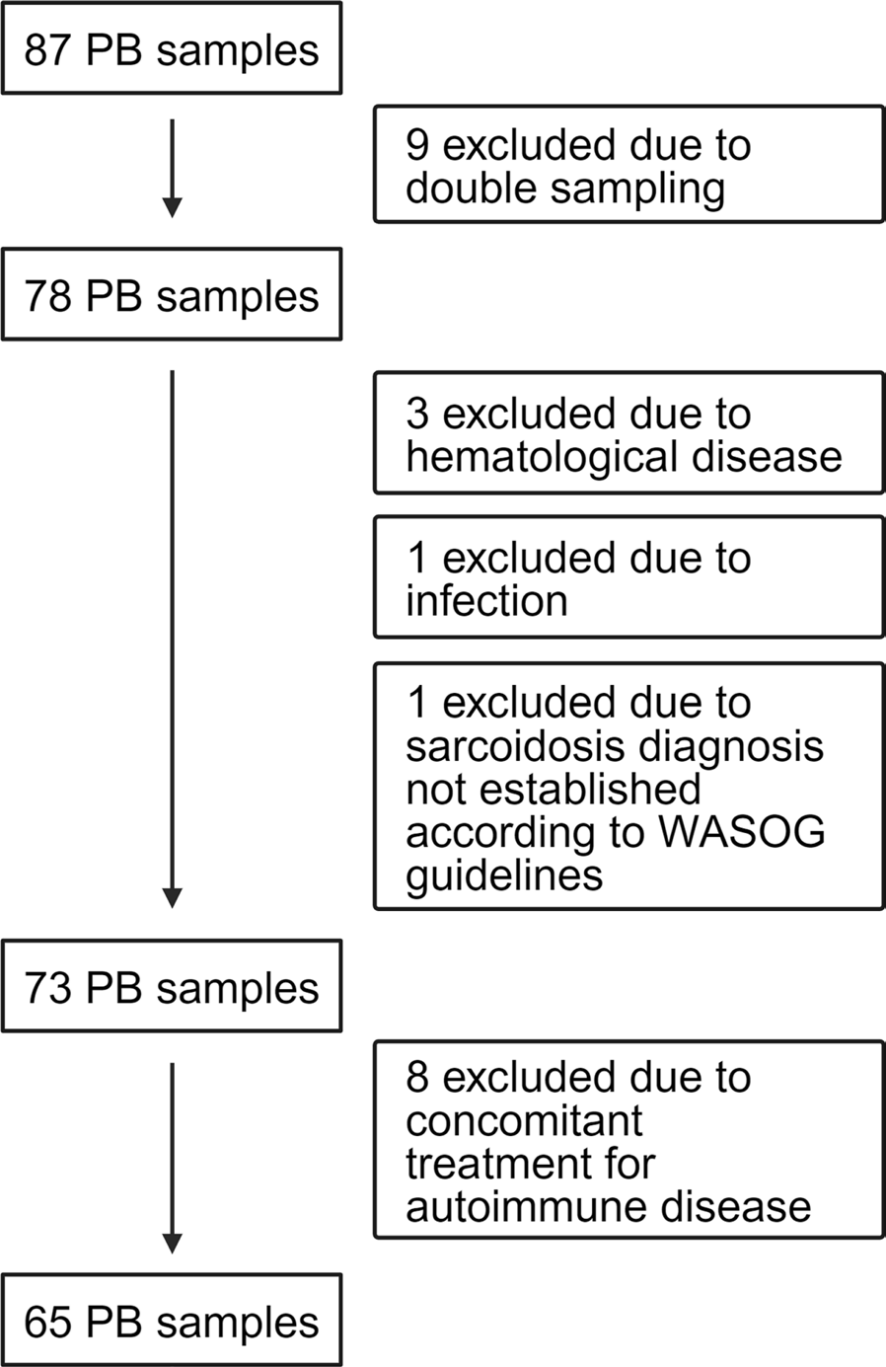
Exclusion process. Nine patients had been seen several times during the study period and thus left several samples. Only the first sample was used for analysis, leaving 78 patients. After applying exclusion criteria 65 remained. WASOG criteria refers to criteria outlined by the World Association of Sarcoidosis and other Granulomatous Disorders, see reference number 19. Data on peripheral blood (PB) lymphocyte subtypes were available from 62, serum tumour necrosis α (s-TNF-α), percent of predicted value for Forced Vital Capacity (FVC) and Forced Expiratory Volume in one second (FEV1) from 59, and diffusion capacity of the lung for carbon monoxide (DLCO) from 57 patients. Four patients were treated with angiotensin convering enzyme (ACE) inhibitors and therefore excluded from analysis of ACE. Data on 8 patients excluded due to concomitant treatment for autoimmune disease are found in Supplement 1

87 blood samples were taken during the study period. After applying exclusion criteria 65 remained, see Fig. 1. Patient characteristics for all 65, and subgroups with and without PB total lymphopenia at inclusion are shown in Table 1. Data on patients excluded due to concomitant treatment for an autoimmune disease are given in Additional file 1. Median number of years from sarcoidosis diagnosis to inclusion was 7 (25th–75th percentile, 4–10). The very first total PB lymphocyte concentration, retrospectively retrieved from the medical chart, was recorded at median 6 (25th–75th percentile, 3–8) years before inclusion. Six patients were then on treatment with immunosuppressants and four had previously been treated but was without treatment at time for PB lymphocyte sampling. For details on these patients, see Additional file 2. Three patients were included at diagnosis and hence, retrospective values for PB total lymphocyte concentrations were not available. These three were excluded when comparing the first available value with that at inclusion.

**Table 1:** Clinical characteristics of patients at inclusion

Parameter	All patients N = 65	Lymphopenia N = 23	No lymphopenia N = 42
Sex (M/F)	40/25	15/8	25/17
Age (years)	55 (48–63)	54 (49–64.5)	58 (45.5–56)
Scadding (0/1/II/III/IV)	9/8/28/8/12	0*/2/15*/1/5	9*/6/13*/7/7
EPM (yes/no)	41/24	15/8	26/16
LS/non-LS/indeterminate	62/2/1	23/0/0	39/2/1
Treated (yes/no)^1^	35/30	17/6*	18/24*
Treatment (no/CS/cytotx ± CS/TNFi + CS/MTX)^1^	30/19/7/9	6*/10/5/2	24*/9/2/7
Resolving + stable/active/treated	18/12/35	2*/4/17*	16*/8/18*
Disease duration (years)^2^	7 (4–10)	7 (4.5–10)	6.5 (3.25–9)
FVC%	88 (78.5–96)	83 (76.5–92.5)	90 (82.3–100)
FEV1%	80 (70–90.5)	77 (68–86)	82 (72–93.3)
DLCO%	81 (71–89)	83 (75–87)	78 (65–89.5)
Elevated ACE (yes/no)	10/51	4/18	6/33
Autoimmune disease (yes/no)	3/65	1/22	2/40

Patients with peripheral (PB) total lymphopenia and normal PB total lymphocytes are denoted Lymphopenia and No lymphopenia, respectively. * denotes significant difference (*p* < 0.05) between the groups. Data are presented as n or median (25th–75th percentile). F = female, M = male, Scadding = radiographic extent of sarcoidosis assessed by chest X-ray using Scadding staging system (0-IV), EPM = extra pulmonary manifestations. FVC%, FEV1% and DLCO% denote percent of predicted value for Forced Vital Capacity, Forced Expiratory Volume in one second and diffusion capacity of the lung for carbon monoxide. Resolving** + **stable/active/treated refers to resolving or stable, active and treated disease, respectively. Disease duration = years with sarcoidosis at inclusion. CS = corticosteroids, cytotox ± CS denotes treatment with methotrexate (MTX) or azathioprine with or without CS, TNFi + CS/MTX denotes treatment with TNF-α inhibitor in combination with CS or MTX

^1^One patient had terminated CS treatment one week before inclusion and one had terminated taking MTX two months before inclusion, both these patients were regarded as treated in analysis

^2^Patients included at diagnosis were classified as having a disease duration of 0 years, and those included within one year from diagnosis were classified as having a disease duration of 0.5 years. One was diagnosed in the 90’s, it was not possible to verify the exact date, duration was estimated to 25 years

### PB total lymphocytes

PB total lymphopenia at inclusion was observed in 23 out of 65 patients and these patients less often disclosed a resolving/stable disease and were more often under treatment than patients with normal PB total lymphocyte concentration (*p* < 0.05 for both).

No difference in lung function parameters was observed but patients with PB total lymphopenia were more often classified into Scadding stage II (*p* < 0.05) while stage 0 was more frequent in patients with normal PB total lymphocyte concentration (*p* < 0.05), see Table 1 for details.

**Fig. 2:**
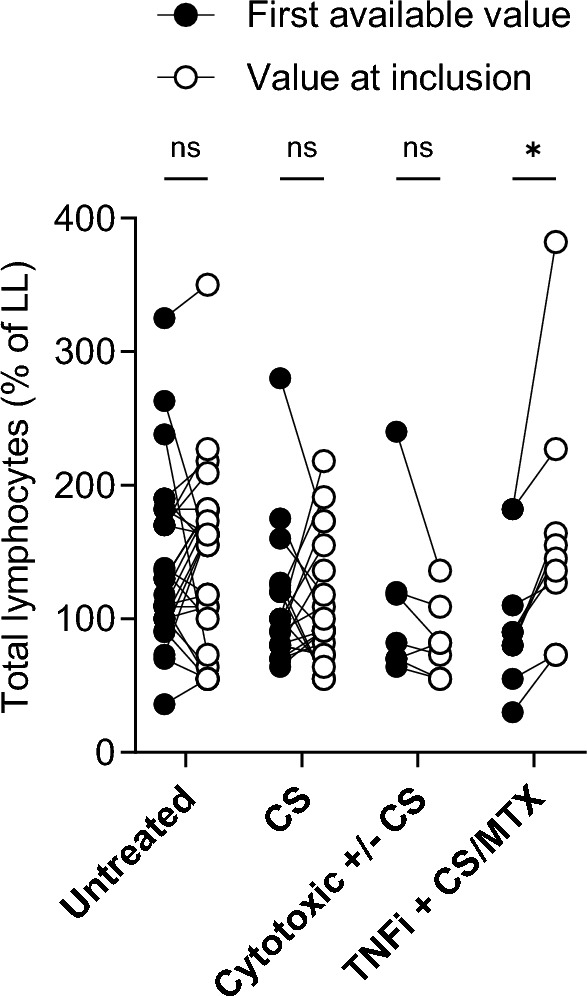
Comparison of first retrospectively identified value of peripheral blood total lymphocyte concentration, expressed as percentage of lower limit of normal (LL), with the value at inclusion in different treatment groups. Untreated (n = 30) denotes patients without treatment at inclusion. CS (n = 19), cytotoxic ± CS (n = 7) and TNFi + CS/MTX (n = 9) denote patients treated with single corticosteroids, single methotrexate (MTX)/azathioprine or in combination with CS, and inhibitors of TNF-α (TNFi) in combination with CS or MTX. *p < 0.05, ns = not significant

PB total lymphocyte concentration was stable over time, median percentage of lower limit was 109% (25th–75th percentile, 80–155) at first retrospective identified value and 118% (25th–75th percentile, 75–164) at inclusion (ns). PB lymphopenia at first measurement associated with a later need för treatment (*p* = 0.005). These analyses were also run excluding the six patients that were treated with immunosuppressants at time for the very first available PB lymphocyte counts but the results did not change, details are described in Supplement 2. CS and cytotoxic ± CS did not influence the value, but patients treated with TNFi + CS/MTX disclosed an increase at inclusion compared to the first retrospectively identified value (*p* < 0.05), see Fig. 2.

### PB lymphocyte subsets

**Fig. 3:**
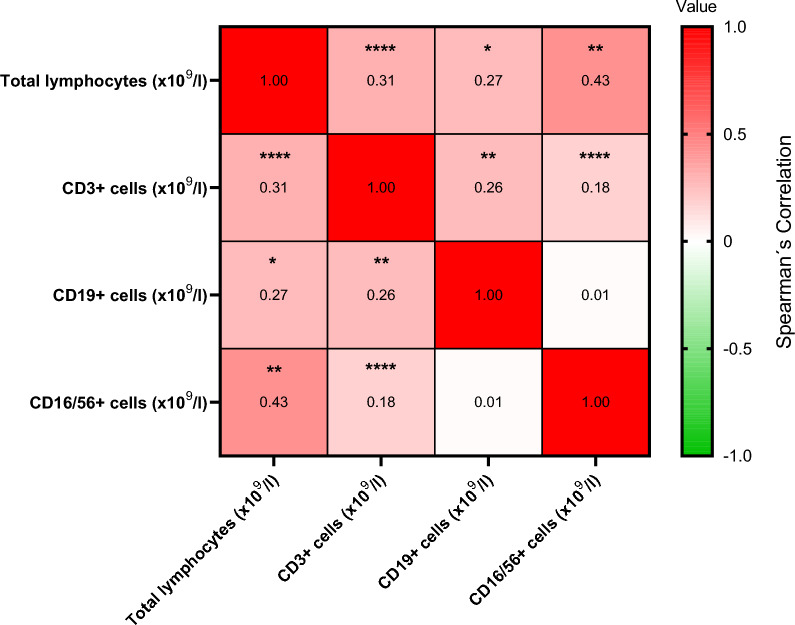
Correlations between peripheral blood lymphocyte subset concentrations. The numbers represent correlation coefficients. *p < 0.05, **p < 0.01, ****p < 0.0001

PB lymphocyte penia were observed to some degree across all subsets, also in the group with normal total lymphocyte counts, shown in Table 2. Median cell concentrations of CD3+ and CD19+ cells below reference values were the most common abnormalities, and present in about one third of all patients. CD19+ cell penia was similarly distributed in both groups (PB total lymphopenia and no total lymphopenia), while CD3+ cell penia was most frequent in the total lymphopenia group (*p* < 0.001), more than 90% of patients had concentrations below reference. CD19+ cell counts were generally low and close to the lower limit of normal in the majority of patients, see Table 3. Percentages of CD19+ and CD16/56+ cells were higher in the PB total lymphopenia group than in the no total lymphopenia group (*p* < 0.05 and 0.001, respectively). As evident from Table 3, this was due to the pronounced reduction of CD3+ cell concentration in the total lymphopenia group. The lymphocyte subsets correlated highly, especially CD3+ cells with total lymphocytes and CD16/56+ cells (*p* < 0.0001 for both), shown in Fig. 3.

**Table 2:** Number of patients with peripheral blood (PB) lymphocyte subtype penias

Parameter	All patients (n = 62)	Lymphopenia (n = 21)	No lymphopenia (n = 41)
CD3+ penia (%)	8 (12.9)	7 (33.3)***	1 (2.4)***
CD3+ penia (×10^9^/l)	21 (33.9)	19 (90.5)***	2 (4.9)***
CD19+ penia (%)	15 (24.2)	3 (14.3)	12 (29.3)
CD19+ penia (×10^9^/l)	23 (37.1)	9 (42.9)	14 (34.1)
CD16/56+ penia (%)	1 (1.6)	0 (0)	1 (2.4)
CD16/56+ penia (×10^9^/l)	7 (11.3)	4 (19.1)	3 (7.3)

Penias were defined as concentrations (×10^9^/l) and percentages (%) below normal lower limit. Lymphopenia and No lymphopenia denote patients with and without PB total lymphopenia, respectively. For 3 out of all 65 patients, data on lymphocyte subsets were missing. Data are presented as n (%). ***p < 0.001 denotes significant difference between groups

**Table 3:** Details on lymphocyte subsets and TNF-α

Parameter	All patients (n = 62)	Lymphopenia (n = 21)	No lymphopenia (n = 41)
CD3+ % (59–83)	71 (65,3–77)	66 (57–71)***	72 (67–79)***
CD3+ × 109/l (0.65–1.57)	0.91 (0.57–1.33)	0.52 (0.4–0.6)***	1.18 (0.90–1.49)***
CD19+ % (6–17)	10 (6–13)	12 (9–16)*	9 (5–11)*
CD19+ × 109/l (0.08–0.28)	0.11 (0.06–0.19)	0.08 (0.05–0.15)	0.12 (0.06–0.20)
CD16/56+ % (6–26)	16.5 (12–22)	19 (15–25)*	15 (11–20)*
CD16/56+ × 109/l (0.1–0.35)	0.21 (0.13–0.30)	0.13 (0.11–0.20)***	0.26 (0.18–0.32)***
TNF-α pg/ml (<20)#	13 (9–21)	12 (10–20)	13 (8–27)

Data is presented for the whole cohort (for 3 out of all 65 patients, data on lymphocyte subsets were missing) and stratified into patients with peripheral blood (PB) total lymphopenia and normal PB total lymphocytes (denoted Lymphopenia and No lymphopenia, respectively). *and ***denote significant difference (*p* < 0.05 and *p* < 0.001, respectively) between those groups. Data are presented as n or median (25th–75th percentile). Values in brackets in the parameter column are reference values. #59 patients were included in analysis of TNF-α, 18 and 41 in lymphopenia and no lymphopenia group, respectively

### PB lymphocyte subsets in relation to disease course and treatment

**Fig. 4:**
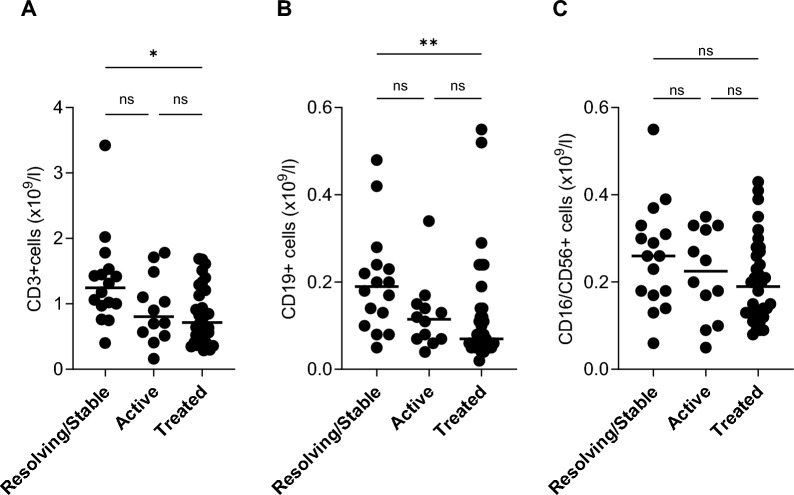
Concentrations of peripheral blood CD3+ (**A**), CD19+ (**B**) and CD16/56+ (**C**) cells in relation to disease course; resolving/stable (n = 16), active (n = 12) and treated (n = 34). Bars represent median values. * and ** denote significant difference (*p* < 0.05 and *p* < 0.01, respectively) between groups, ns = not significant

**Fig. 5:**
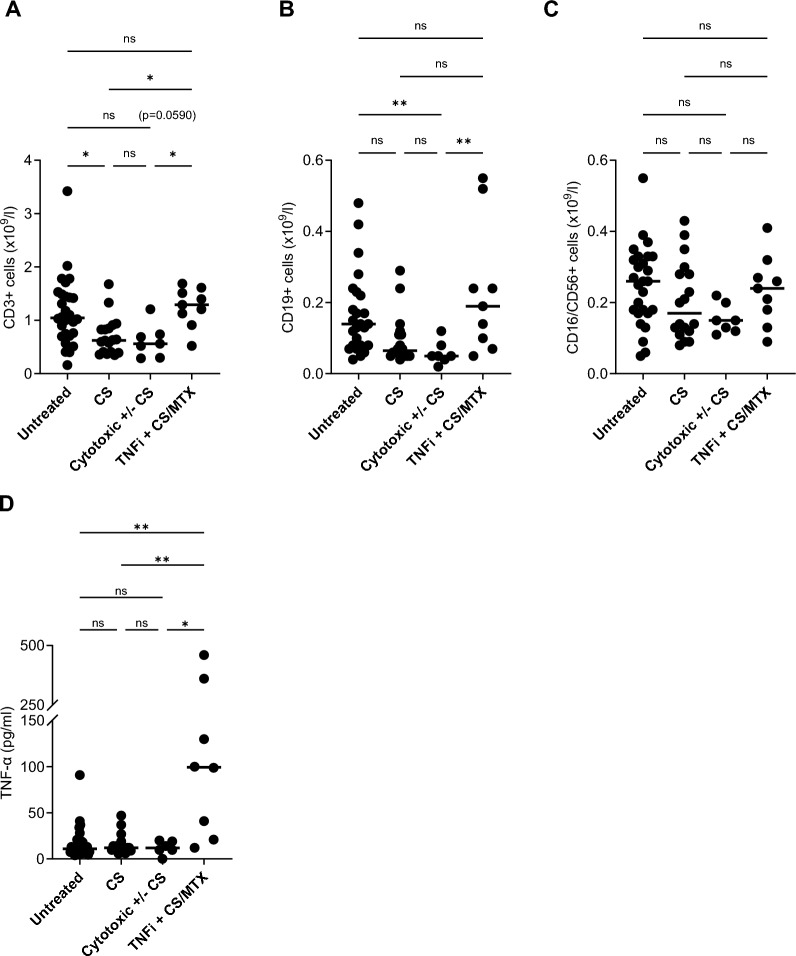
Concentrations of peripheral blood CD3+ (**A**), CD19+ (**B**), CD16/56+ (**C**) cells and serum TNF-α (**D**) in relation to treatment status. Untreated (n = 28 in **A**–**C** and 29 in **D**) denotes patients without treatment at inclusion. CS (n = 18 in **A**–**C** and 16 in **D**), cytotoxic ± CS (n = 7 in **A**–**C** and 6 in **D**) and TNFi + CS/MTX (n = 9 in **A**–**C** and 8 in **D**) denote patients treated with single corticosteroids, single methotrexate (MTX)/azathioprine or in combination with CS, and inhibitors of TNF-α (TNFi) in combination with CS or MTX. For the TNFi + CS/MTX group TNF-α evels were only available from 8 out of 9 patients. * and ** denote significant difference (*p* < 0.05 and *p* < 0.01, respectively) between groups, ns = not significant

Median CD3+, CD19+ and CD16/56+ cell concentrations were highest in patients with stable/resolving, lower in patients with active disease and lowest in treated patients, details are shown in Fig. 4A–C. Among treated patients, the median cell concentrations for lymphocyte subsets varied between treatments. Patients treated with TNFi + CS/MTX revealed median concentrations similar to those of untreated, whereas patients receiving CS and cytotoxic ± CS exhibited the lowest concentrations, see Fig. 5A–C.

CD3+ cell concentrations were lower in Scadding stage II (0.74 × 10^9^/l, 25th–75th percentile 0.52–0.94) and higher in 0 (1.43 × 10^9^/l, 25th–75th percentile 1.18–1.49) compared to the other stages (*p* < 0.05 for both).

### TNF-α

The concentrations of s-TNF-α differed based on the treatment with patients receiving TNFi + CS/MTX exhibiting the highest levels, see Fig. 5D. S-TNF-α concentrations did not correlate with any of the other examined parameters, i.e. PB total lymphocyte, CD3+, CD19+ and CD16/56+ cell concentrations and percentages, disease course, gender, Scadding stage, lung function parameters, s-ACE and EPM.

## Discussion

In this study we sought to understand the association between PB lymphopenia with poor prognosis and unsatisfactory responses to conventional treatments in sarcoidosis [7, 8, 12, 23, 24]. For this we mapped the proportions of PB lymphocyte subsets, s-TNF-α levels and correlated these metrics with clinical parameters.

PB total lymphopenia was found in 35% of patients, associated with a treatment-requiring disease and involved CD3+, CD19+ and CD16/56+ cells, putatively representing T-, B- and NK cells, respectively. Lymphocyte subtype penias were also observed in patients with normal total lymphocyte counts with CD19+ cell penia being the most frequent abnormality, observed in 34% of patients with normal counts. In contrast, CD3+ cell penia was infrequently seen in this group, yet it was present in over 90% of patients with PB total lymphopenia. Thus, CD3+ cell penia seems to be a feature, distinguishing patients with a treatment-requiring disease from those with a stable/resolving disease, a finding in line with previous reports linking sarcoidosis associated T-cell penia to disease activity, high inflammation and progressive disease [7, 24, 25].

The majority of patients with PB total lymphopenia were treated, and treatment was associated with lower CD3+ and CD19+ cell counts. As the treated group included patients with cytotoxic therapy it can be argued that lymphopenia is caused by treatment. However, the total lymphocyte concentration did not change from the first retrospectively identified value, recorded when the patients were untreated, to the value recorded at inclusion when treatment had been initiated. The TNFi treated patients made up an exception, they showed an increase in PB lymphocyte concentration despite concomitant treatment with CS or cytotoxic agents. Therefore, we believe that the lymphopenia is not related to treatment per se, rather it relates to a progressive disease and need for treatment, a hypothesis supported by previous findings by us and others [5, 6, 23, 26].

The assertion that sarcoidosis PB lymphopenia results from migration of lymphocytes into sites with active inflammation is challenged by our findings, which revealed no association with EPM. However, we did not actively screen for EPM which might have led to an under estimation. Furthermore, we think our finding that patients without visible changes on chest X-ray more often had normal total PB total lymphocyte counts and higher CD3+ cell counts compared to patients in the other Scadding stages, while patients with both lymph node enlargement and parenchymal infiltrates disclosed lower PB total lymphocyte and CD3+ cell counts, can speak for the theory of PB lymphopenia being due to a migration of lymphocytes to an inflamed lung.

CD16/56+ cell penia was less frequent than T- and B-cell penia, but PB total lymphocytes, CD3+ and CD16/56+ cell counts correlated highly, suggesting a tight connection between these subsets. Interestingly, a very recent publication reported that fibrotic sarcoidosis was associated with higher percentages of BALF NK cells than the other stages, and the authors suggested that these cells are linked to fibrosis development [27]. In this study we did not find any correlation with fibrosis and lymphopenia. However, we think that may be due to few observations as we in a previous study including more patients found an association between PB total lymphopenia and fibrosis [12].

Our results are also in line with the paradigm positing that PB lymphopenia in sarcoidosis results from an imbalance in immunoregulatory functions [9, 23, 28–30]. If binding of TNF-α to T_regs_ is inhibited by TNFi, the T_reg_ antiproliferative function thought to be mediated by TNF-α, could be reversed/diminished, and thus provide conditions for expansion of the lymphocyte pool [9, 31, 32] and explain the increased PB total lymphocyte and CD3+ cell counts in TNFi treated patients, an observation also made by others [7, 33]. Therefore, we were surprised that the levels of s-TNF-α were generally low, without association to neither PB total lymphocyte and CD3+ cell counts, nor parameters reflecting disease activity and severity. Also, in a previous study from our group, low concentrations of s-TNF-α were detected and the levels did not differ from those of healthy controls [34]. Low s-TNF-α levels in sarcoidosis patients were also reported in a novel publication from India by Jain et al*.* [35]. Contrasting with these findings, are results from an American study showing that elevated levels of s-TNF-α were linked to more severe disease outcomes. That study included also patients of African-American ancestry, which seem to have higher s-TNF-α levels compared to patients of European ancestry [36] and one study from Japan found s-TNF-α concentrations being higher in sarcoidosis patients compared to healthy controls [37]. Thus, the conflicting results on s-TNF-α levels may depend on ethnic differences between sarcoidosis patients included in different studies. Interestingly, a Dutch study reported that sarcoidosis patients, mainly of Caucasian ancestry, expressed higher levels of TNF receptor 2 (TNFR2) on T_regs_ than healthy controls and the expression, as well as concentration of soluble TNFR2 in PB, decreased following TNFi treatment [2, 38]. These results and our observation that patients receiving TNFi treatment disclosed the highest concentrations of s-TNF-α, a finding also reported from other researchers [2], suggest that serum levels of TNF-α alone may not explain the disease course in all sarcoidosis patients across different populations. Rather, we believe that different expression of regulatory receptors as well as local release of TNF-α, perhaps not reaching the systemic circulation, from for instance mononuclear phagocytes, i.e. potent producers of TNF- α and capable of activating T-cells, may account for the effects of TNF- α in sarcoidosis [34, 38, 39].

An increasing body of evidence indicate also B cells as important factors in sarcoidosis pathogenesis [40]. We found that B-cell counts were generally low and B-cell penia was the most frequent abnormality of the lymphocyte subsets investigated. The altered distribution of B-, as well as other lymphocyte subsets reported here, resemble the PB lymphocyte profile in several autoimmune disorders [14, 40–42]. For instance, it was recently reported that patients with inflammatory myositis disclosed PB total, T-, B and NK- cell penia, and patients with higher disease activity encountered lower total lymphocyte counts [43], i.e. a pattern similar to what we report here for sarcoidosis patients. In this context, it is interesting that B-cells are emerging as pivotal players in several autoimmune diseases of which many coexist with sarcoidosis, and can be treated with TNFi, suggesting shared pathogenetic mechanisms [43, 44]. Together, these findings emphasize the need for comparative studies on sarcoidosis and autoimmune diseases for insights into the imbalance in lymphocyte populations linked to the development of these diseases.

Strengths of this study include a well-defined patient group, composed of mainly white ancestry individuals of Nordic descent, all well characterized phenotypically with a long observation time. All data was collected by a specialist in respiratory medicine, which should validate the diagnosis further.

Besides the already mentioned limitation about not actively screening for EPM, our study has some other limitations. As Karolinska University Hospital is a referral center, our data likely has a bias toward more severe cases. We only had long term data on PB total lymphocyte, not subset counts, and the numbers of included patients were too small to allow for comparison between groups of different subset penia combinations. Also, the administrated doses of immunosuppressants varied but the numbers of included patients were too small to allow for analysis of possible dose-dependent effects. While we consider the uniform ethnic composition of our cohort to be a strength, it may also pose a limitation, as the findings may not be easily applicable to other ethnic groups.

## Conclusions

To conclude, B-cell penia was encounterad in about one third of patients with sarcoidosis, also in patients with normal PB total lymphocyte counts, while T-cell penia was more frequent in patients with PB total lymphopenia and treatment-requiring disease. Sarcoidosis patients with PB lymphopenia at diagnosis should be carefully monitored as they are at risk of developing a non-resolving disease and need for treatment. TNFi, but not CS and cytotoxic treatments seem to increase PB total lymphocyte, CD3+ and CD19+ cell counts, indicating that patients with PB lymphopenia might benefit from early intervention with TNFi. Finally, the immune cell alterations reported here are some of the features linking sarcoidosis to autoimmune diseases, emphasizing the need for future studies on common mechanisms in development of these diseases.

## Supplementary Information

Additional file 1.

Additional file 2.

## Data Availability

The datasets used and/or analysed during the current study are available from the corresponding author on reasonable request.

## Abbreviations

LS: Löfgren’s syndrome
Non-LS: Non-Löfgren’s syndrome
TNF-α: Tumour necrosis factor α
PB: Peripheral blood
CS: Corticosteroids
MTX: Methotrexate
T_regs_: T regulatory cells
NK cells: Natural killer cells
WASOG: World Association of Sarcoidosis and other Granulomatous Disorders
s-ACE: Serum angiotensin-converting enzyme
TNFi: TNF-α inhibitor
EPM: Extra pulmonary manifestations
