# Supplementary material for: Associations of peripheral blood lymphopenia to disease course, treatment and TNF-α in sarcoidosis
Source: Respir Res. 2025 Apr 9;26:130. doi: 10.1186/s12931-025-03212-x (PMC11983878; doi:10.1186/s12931-025-03212-x)
Supplement: Supplementary file 1 — Additional file 1. [file 12931_2025_3212_MOESM1_ESM.docx]

**Additional file 1.** Details on sarcoidosis patients excluded from analysis due to concomitant treatment for an autoimmune disease.

| **Parameter** | **Value** |
| --- | --- |
| **IBD/AS/PA/SS/PSC** | 4/3/1/1/1 |
| **Sex (male/female)** | 5/3 |
| **Age (years)** | 49.5 (42.5-51.5) |
| **Scadding (0/I/II/III/IV)** | 0/1/2/4/1 |
| **Extra pulomonary manifestations (yes/no)** | 5/3 |
| **Löfgren´s syndrome/non-Löfgren´s syndrome** | 0/8 |
| **Treated (yes/no)** | 8/0 |
| **Treatment (asacol/secunikumab/cytotoxic±CS/TNFi±CS)** | 4/1/4/4 |
| **Disease duration (years)** | 4.5 (3-7.5) |
| **FVC%** | 92.5 (76-95.3) |
| **FEV1%** | 82.5 (66.5-90.3) |
| **DLCO%** | 80.5 (65.8-107) |
| **Total lymphopenia (yes/no)** | 4/4 |
| **CD3+ penia (yes/no)** | 3/5 |
| **CD19+ penia (yes/no)** | 1/7 |
| **CD16/56+ penia (yes/no)** | 1/7 |
| **Total lymphocytes x10^9^/l (1.1-3.5) #** | 1.0 (0.8-1.40) |
| **Total lymphocytes x10^9^/l, percentage of lower limit** | 100 (73-150) |
| **CD3+ x10^9^/l (0.65-1.57)** | 0.73 (0.51-0.89) |
| **CD3+% (59-83)** | 67 (63.3-71.3) |
| **CD19+ x10^9^/l (0.08-0.28)** | 0.12 (0.1-0.16) |
| **CD19+% (6-17)** | 13 (9-18.5) |
| **CD16/56+ x10^9^/l (0.1-0.35)** | 0.15 (0.10-0.22) |
| **CD16/56+% (6-26)** | 18.5 (10.5-22.3) |
| **S-TNF-α pg/ml (<20)^** | 13 (8.4-145) |

Data are presented as n or median (25^th^ – 75^th^ percentile). Values in brackets in the parameter column are reference values (ref values). IBD/AS/PA/SD/PSC refers to type of autoimmune disease; inflammatory bowel disease/ankylosing spondylitis/psoriatic arthritis/Sjögren´s syndrome/primary sclerosing cholangitis. Autoimmune diseases co-existed and some patients were treated with several drugs, therefore the number of patients referring to type of autoimmune disease and treatment exceeds 8. Scadding=radiographic extent of sarcoidosis assessed by chest X-ray using Scadding staging system (0-IV). CS=corticosteroids, cytotoxic denotes patients treated with methotrexate or azathioprine, TNFi refers to patients treated with TNF-α inhibitors (infliximab, adalimumab, etanercept). Disease duration=years with sarcoidosis at inclusion. Total lymphopenia, CD3+, CD19+ and CD16/56+ penia denote number of patients with concentrations (x10^9^/l) below lower limit of normal. All values for immune cells refer to peripheral blood. FVC%, FEV1% and DLCO% denotes percent of predicted value for Forced Vital Capacity, Forced Expiratory Volume in one second and diffusion capacity of the lung for carbon monoxide.

#7 patients were included in analysis of median total lymphocyte concentration as the sample from one patient was analyzed at an external laboratory with different reference values. Therefore percentage of lower normal limit was also used for calculating median concentration of total lymhocytes, all 8 patients were included in that analysis.

^7 patients were included in analysis of serum (s)-TNF-α.
