# Supplementary material for: Associations of peripheral blood lymphopenia to disease course, treatment and TNF-α in sarcoidosis
Source: Respir Res. 2025 Apr 9;26:130. doi: 10.1186/s12931-025-03212-x (PMC11983878; doi:10.1186/s12931-025-03212-x)
Supplement: Supplementary file 2 — Additional file 2. [file 12931_2025_3212_MOESM2_ESM.docx]

**Additional file 2.** Data on treatment status at first retrospectively identified PB total lymphocyte value.

Six patients were treated with immunosuppressants at first retrospectively identified value, five with single corticosteroids (CS) and of these two were still treated with CS at inclusion and three were without any treatment. One patient was treated with CS and methotrexate (MTX) at first retrospectively identified value, at inclusion this patient was still treated with CS but with TNF-α inhibitor (TNFi) instead of MTX. Analysis comparing the very first value for PB lymphocyte concentration with that at inclusion described in the Results section was performed including these six patients. We also ran the analysis excluding these six and got similar results; 109% (25^th^-75^th^ percentile, 81-144) at first retrospective identified value and 118% (25^th^-75^th^ percentile, 80-163) at inclusion (ns). Analysis of treatment at inclusion in relation to lymphopenia at first retrospectively identified value was also run with these six patients excluded and the results was similar to that described in the Result section including these six. Lymphopenia at first identified value associated with treatment at inclusion (p=0.003).

Four patients had previously been treated with CS but was terminated 2, 10, 13 and 19 months, respectively, before the very first recorded value. These patients are included in all analyses.
